# Supplementary material for: Tissue sodium excess is not hypertonic and reflects extracellular volume expansion
Source: Nat Commun. 2020 Aug 24;11:4222. doi: 10.1038/s41467-020-17820-2 (PMC7445299; doi:10.1038/s41467-020-17820-2)
Supplement: Supplementary file 1 — Supplementary Information [file 41467_2020_17820_MOESM1_ESM.docx]

**Tissue Sodium Excess is not Hypertonic and Reflects ECV Expansion**

Giacomo Rossitto, et al.

**SUPPLEMENTARY MATERIAL**

**SUPPLEMENTARY TABLES (1-5)** pag. 2

**SUPPLEMENTARY FIGURES (1-9)** pag. 7

**SUPPLEMENTARY NOTE I** pag. 19

**SUPPLEMENTARY NOTE II** pag. 20

**SUPPLEMENTARY NOTE III** pag. 22

**SUPPLEMENTARY REFERENCES** pag. 22

**Supplementary Table 1. Arterial myography – effect of in-vivo High Na^+^ (HS diet)**

|  |  |  | **n** | **WKY - NS** | **n** | **SHRSP - NS** | p vs  WKY-NS | **n** | **WKY - HS** | p vs  WKY-NS | **n** | **SHRSP - HS** | p vs  SHRSP-NS |
| --- | --- | --- | --- | --- | --- | --- | --- | --- | --- | --- | --- | --- | --- |
| **ALL** | **U4** | **Max con** | **16** | **79.20 (75.31 to 83.35)** | **16** | **92.68 (88.63 to 96.91)** | <0.001 | **16** | **72.81 (68.74 to 77.19)** | 0.032 | **17** | **93.15 (87.81 to 99.02)** | 0.893 |
|  |  | **LogEC50** |  | **-7.238 (-7.312 to -7.163)** |  | **-7.361 (-7.428 to -7.292)** | 0.017 |  | **-7.317 (-7.405 to -7.226)** | 0.180 |  | **-7.274 (-7.367 to -7.178)** | 0.142 |
|  | **SNP** | **Max rel** | **13** | **7.57 (2.68 to 12.11)** | **12** | **5.12 (1.19 to 8.85)** | 0.426 | **16** | **13.24 (7.73 to 18.25)** | 0.119 | **16** | **10.21 (6.99 to 13.29)** | 0.041 |
|  |  | **LogIC50** |  | **-7.868 (-8.003 to -7.725)** |  | **-8.052 (-8.164 to -7.937)** | 0.043 |  | **-7.776 (-7.926 to -7.614)** | 0.393 |  | **-7.656 (-7.742 to -7.568)** | <0.001 |
| Male | U4 | Max con | 8 | 72.23 (67.18 to 77.66) | 6 | 84.93 (80.53 to 89.55) | <0.001 | 8 | 67.09 (62.53 to 71.92) | 0.144 | 7 | 89.95 (80.66 to 101.30) | 0.330 |
|  |  | LogEC50 |  | -7.295 (-7.399 to -7.186) |  | -7.641 (-7.722 to -7.562) | <0.001 |  | -7.258 (-7.359 to -7.159) | 0.624 |  | -7.283 (-7.463 to -7.082) | <0.001 |
|  | SNP | Max rel | 8 | 6.88 (-0.47 to 13.37) | 5 | 5.86 (-1.39 to 12.40) | 0.839 | 7 | 18.24 (7.20 to 26.91) | 0.059 | 6 | 8.52 (4.23 to 12.60) | 0.490 |
|  |  | LogIC50 |  | -7.861 (-8.057 to -7.646) |  | -7.894 (-8.09 to -7.686) | 0.822 |  | -7.709 (-7.989 to -7.366) | 0.386 |  | -7.550 (-7.655 to -7.443) | 0.002 |
| Female | U4 | Max con | 8 | 86.21 (80.65 to 92.37) | 10 | 96.38 (92.45 to 100.40) | 0.005 | 8 | 77.16 (71.08 to 84.05) | 0.044 | 10 | 95.20 (88.88 to 102.20) | 0.766 |
|  |  | LogEC50 |  | -7.190 (-7.287 to -7.090) |  | -7.212 (-7.270 to -7.156) | 0.688 |  | -7.370 (-7.501 to -7.228) | 0.036 |  | -7.269 (-7.371 to -7.163) | 0.343 |
|  | SNP | Max rel | 5 | 8.53 (2.07 to 14.47) | 7 | 4.47 (3.23 to 8.68) | 0.273 | 9 | 9.29 (3.08 to 14.95) | 0.868 | 10 | 11.36 (6.86 to 15.56) | 0.029 |
|  |  | LogIC50 |  | -7.875 (-8.051 to -7.690) |  | -8.162 (-8.290 to -8.031) | 0.008 |  | -7.821 (-7.982 to -7.647) | 0.678 |  | -7.729 (-7.853 to -7.602) | <0.001 |

U4 = U46619, thromboxane A2 receptor agonist; SNP = sodium nitroprussiate, nitric oxide (NO) donor. Max con and rel = maximal contraction (% of response to 62.5 mM KCl) and relaxation (% of pre-constriction with U46619 concentration producing 75±5% of maximal contractile response), respectively. LogEC_50_/IC_50_ = Log of half maximal effective/inhibitory concentrations, respectively. Data are presented as non-linear least-square regression estimates (95%CI). Numbers of viable vessels (n) available for analysis are listed in the upper left corner of each group/experimental condition. Source data are provided as a Source Data file.

**Supplementary Table 2. Arterial myography – effect of ex-vivo hypertonic Na^+^ (+15 mmol L^-1^; HT)**

|  |  |  | **n** | **WKY-NS** | **n** | **SHRSP-NS** | p vs  WKY-NS | **n** | **WKY-NS + HT** | p vs  WKY-NS | **n** | **SHRSP-HS +HT** | p vs  SHRSP-NS |
| --- | --- | --- | --- | --- | --- | --- | --- | --- | --- | --- | --- | --- | --- |
| **ALL** | **U4** | **Max con** | **12** | **87.52 (83.84 to 91.40)** | **11** | **90.95 (85.30 to 97.39)** | 0.317 | **11** | **84.88 (80.41 to 89.78)** | 0.382 | **12** | **95.21 (89.49 to 101.5)** | 0.328 |
|  |  | **LogEC50** |  | **-7.717 (-7.796 to -7.636)** |  | **-7.568 (-7.689 to -7.438)** | 0.039 |  | **-7.674 (-7.776 to -7.568)** | 0.516 |  | **-7.553 (-7.663 to -7.436)** | 0.856 |
|  | **SNP** | **Max rel** | **11** | **5.95 (0.67 to 10.84)** | **9** | **6.33 (0.92 to 11.19)** | 0.924 | **11** | **4.55 (-0.60 to 9.39)** | 0.697 | **9** | **7.63 (2.92 to 12.10)** | 0.711 |
|  |  | **LogIC50** |  | **-8.004 (-8.152 to -7.848)** |  | **-7.334 (-7.467 to -7.194)** | <0.001 |  | **-8.299 (-8.449 to -8.142)** | 0.007 |  | **-7.891 (-8.014 to -7.762)** | <0.001 |
| Male | U4 | Max con | 8 | 87.43 (83.08 to 92.08) | 6 | 88.26 (82.31 to 94.87) | 0.823 | 7 | 88.27 (83.12 to 93.91) | 0.808 | 6 | 86.46 (79.91 to 93.83) | 0.697 |
|  |  | LogEC50 |  | -7.746 (-7.842 to -7.646) |  | -7.717 (-7.847 to -7.578) | 0.716 |  | -7.822 (-7.939 to -7.697) | 0.327 |  | -7.639 (-7.784 to -7.487) | 0.437 |
|  | SNP | Max rel | 6 | 7.06 (-1.50 to 14.37) | 5 | 5.45 (-2.75 to 12.59) | 0.778 | 6 | 6.96 (-1.12 to 14.12) | 0.986 | 5 | 10.86 (4.43 to 16.77) | 0.262 |
|  |  | LogIC50 |  | -7.883 (-8.119 to -7.623) |  | -7.387 (-7.580 to -7.178) | 0.003 |  | -8.520 (-8.758 to -8.253) | <0.001 |  | -7.622 (-7.782 to -7.451) | 0.068 |
| Female | U4 | Max con | 4 | 87.48 (80.62 to 95.06) | 5 | 94.72 (84.57 to 108.60) | 0.263 | 4 | 77.34 (74.17 to 80.73) | 0.019 | 6 | 103.90 (95.07 to 114.4) | 0.243 |
|  |  | LogEC50 |  | -7.705 (-7.868 to -7.532) |  | -7.382 (-7.587 to -7.131) | 0.026 |  | -7.451 (-7.518 to -7.379) | 0.011 |  | -7.481 (-7.636 to -7.310) | 0.463 |
|  | SNP | Max rel | 5 | 3.39 (-2.83 to 9.23) | 4 | 7.18 (0.22 to 13.10) | 0.464 | 5 | 2.758 (-2.920 to 8.174) | 0.877 | 4 | 3.44 (-0.19 to 6.98) | 0.306 |
|  |  | LogIC50 |  | -8.124 (-8.282 to -7.957) |  | -7.260 (-7.425 to -7.080) | <0.001 |  | -8.101 (-8.248 to -7.947) | 0.831 |  | -8.191 (-8.283 to -8.099) | <0.001 |

U4 = U46619, thromboxane A2 receptor agonist; SNP = sodium nitroprussiate, nitric oxide (NO) donor. Max con and rel = maximal contraction (% of response to 62.5 mM KCl) and relaxation (% of pre-constriction with U46619 concentration producing 75±5% of maximal contractile response), respectively. LogEC_50_/IC_50_ = Log of half maximal effective/inhibitory concentrations, respectively. Data are presented as non-linear least-square regression estimates (95%CI). Numbers of viable vessels (n) available for analysis are listed in the upper left corner of each group/experimental condition. Source data are provided as a Source Data file.

**Supplementary Table 3. Characteristics of patients.**

| **Variables** | **ALL** | **FEMALES** | **p** | **MALES** |
| --- | --- | --- | --- | --- |
| **Females** | **36 (47.4%)** | - |  | - |
| **Age** (years) | **58 ± 15** | 56 ± 16 | 0.359 | 59 ± 13 |
| **BMI** (kg/m^2^) | **30.3 (27.5-36.3)** | 31.6 (26.5-38.3) | 0.212 | 29.7 (27.8-33.9) |
| **Office SBP** (mmHg) | **148 ± 21** | 148 ± 26 | 0.869 | 149 ± 15 |
| **Office DBP** (mmHg) | **89 ± 12** | 87 ± 13 | 0.278 | 90 ± 11 |
| **Office HR** (beats per minute) | **74 (65-87)** | 77 (66-89) | 0.82 | 73 (64-84) |
| **Uncontrolled HTN** | **56 (73.7%)** | 21 (60%) | **0.006** | 35 (87.5%) |
| **Number of anti-HTN medications** | **2 (0-4)** | 2 (1-3) | **0.011** | 3 (1-3) |
| **0** | **8 (10.5%)** | 6 (16.7%) | 0.056 | 2 (5%) |
| **1** | **17 (22.4%)** | 9 (25%) |  | 8 (20%) |
| **2** | **15 (19.7%)** | 10 (27.8%) |  | 5 (12.5%) |
| **3** | **22 (28.9%)** | 6 (16.7%) |  | 16 (40%) |
| **≥** **4** | **14 (18.4%)** | 5 (13.9%) |  | 9 (22.5%) |
| **ACEi/ARB** | **60 (78.9%)** | 25 (69.4%) | 0.054 | 35 (87.5%) |
| **CCB** | **41 (53.9%)** | 15 (41.7%) | **0.042** | 26 (65%) |
| **Diuretic** | **33 (43.4%)** | 12 (33.3%) | 0.092 | 21 (52.5%) |
| **BB** | **18 (23.7%)** | 7 (19.4%) | 0.41 | 11 (27.5%) |
| **MRA** | **9 (11.8%)** | 2 (5%) | 0.108 | 7 (17.5%) |
| **AB** | **12 (15.8%)** | 6 (5.6%) | 0.842 | 6 (15%) |
| **Obesity** | **41 (53.9%)** | 22 (61.1%) | 0.235 | 19 (47.5%) |
| **Diabetes mellitus** | **10 (13.3%)** | 3 (8.6%) | 0.256 | 7 (17.5%) |
| **Dyslipidaemia** | **43 (58.1%)** | 17 (50%) | 0.192 | 26 (65%) |
| **Chronic kidney disease** | **8 (10.5%)** | 3 (8.3%) | 0.555 | 5 (12.5%) |
| **Na^+^ intake (questionnaire; g/d)** | **2.79 (2.24-3.76)** | 2.64 (2.39-3.44) | 0.323 | 3.09 (2.16-4.56) |
| **p-Na^+^** | **140 (139-142)** | 140 (139-142) | 0.565 | 140 (139-141) |
| **p-Urea (mmol/l)** | **5.3 (4.5-6.5)** | 4.8 (3.9-5.9) | 0.005 | 5.6 (4.9-7.3) |
| **p-Creatinine (umol/l)** | **73 (63-86)** | 65 (55-73) | <0.001 | 84 (72-96) |
| **u-ACR (mg/gCr)** | **6.7 (2.6-16.8)** | 6 (3.4-11.7) | 0.848 | 6.7 (2.3-24.7) |
| **NT-pro-BNP (pg/ml)** | **71.8 (43.1-184.1)** | 79 (46-142) | 0.577 | 68 (34-253) |

Qualitative data presented as n (%) and compared by χ2 test. Quantitative data presented as mean ± SD or median (interquartile range) and compared by Student t-test or Mann-Whitney test, respectively, as appropriate. All tests are two-tailed. BMI = Body Mass Index. SBP = systolic blood pressure. DBP = diastolic blood pressure. HR = heart rate. HTN = hypertension. Uncontrolled HTN = SBP ≥ 140 and/or DBP ≥ 90 mmHg. ACEi/ARB = ACE inhibitors or Angiotensin receptor blockers. CCB = calcium channel blockers. BB = beta blockers. MRA = mineralocorticoid antagonists. U-ACR = urinary albumin to creatinine ratio (random urine). Source data are provided as a Source Data file.

**Supplementary Table 4. Skin histochemical differences between hypertensive males and females.**

|  |  | **MALES**  (n=40) | p vs females | **FEMALES** (n=36) | | | |
| --- | --- | --- | --- | --- | --- | --- | --- |
|  | **Variables** |  |  | **ALL** | pre-menop  (n=14) | p | post-menop  (n=22) |
| Epidermis/superficial dermis (ESD) | **Water content** (mg/mgDW) | **2.57 ± 0.32** | 0.864 | **2.58 ± 0.29** | 2.50 ± 0.21 | 0.244 | 2.62 ± 0.33 |
|  | **Water content** (%WW) | **71.62 ± 2.35** | 0.708 | **71.83 ± 2.36** | 71.32 ± 1.64 | 0.337 | 72.13 ± 2.68 |
|  | **Na^+^ content** (mmol/gDW) | **0.290 ± 0.049** | 0.821 | **0.290 ± 0.044** | 0.264 ± 0.026 | **0.005** | 0.302 ± 0.047 |
|  | **Na^+^ concentration** (mmol/l) | **113.5 ± 8.3** | 0.371 | **111.7 ± 8.9** | 105.8 ± 6.4 | **0.002** | 115.1 ± 8.4 |
|  | **K^+^ content** (mmol/gDW) | **0.076 ± 0.013** | 0.602 | **0.078 ± 0.009** | 0.082 ± 0.009 | **0.032** | 0.075 ± 0.009 |
|  | **K^+^ concentration** (mmol/l) | **29.9 ± 5.5** | 0.694 | **30.4 ± 4.2** | 32.9 ± 3.9 | **0.005** | 28.9 ± 3.7 |
|  | **Na^+^/K^+^ ratio** | **3.93 ± 0.97** | 0.415 | **3.76 ± 0.72** | 3.27 ± 0.50 | **0.001** | 4.06 ± 0.68 |
| Deep dermis  (DD) | **Water content** (mg/mgDW) | **1.68 ± 0.41** | **0.007** | **1.40 ± 0.42** | 1.24 ± 0.26 | 0.159 | 1.46 ± 0.46 |
|  | **Water content** (%WW) | **61.67 ± 6.82** | **0.009** | **57.26 ± 7.02** | 54.69 ± 5.53 | 0.207 | 58.12 ± 7.45 |
|  | **Na^+^ content** (mmol/gDW) | **0.208 ± 0.057** | **<0.001** | **0.158 ± 0.054** | 0.136 ± 0.042 | 0.144 | 0.166 ± 0.056 |
|  | **Na^+^ concentration** (mmol/l) | **122.7 ± 8.9** | **0.004** | **114.0 ± 13.4** | 111.8 ± 9.5 | 0.735 | 114.3 ± 15.1 |
|  | **K^+^ content** (mmol/gDW) | **0.029 ± 0.008** | **0.007** | **0.024 ± 0.006** | 0.022 ± 0.007 | 0.446 | 0.024 ± 0.006 |
|  | **K^+^ concentration** (mmol/l) | **17.2 ± 3.8** | 0.795 | **17.4 ± 3.5** | 17.9 ± 2.6 | 0.449 | 17.1 ± 3.9 |
|  | **Na^+^/K^+^ ratio** | **7.48 ± 1.78** | 0.061 | **6.69 ± 1.65** | 6.15 ± 1.24 | 0.226 | 6.95 ± 1.84 |

Data presented as mean ± SD and compared by two-tailed Student t-test. SBP = systolic blood pressure. Missing data because of technical issues or unavailable sample ≤ 8/total 76 subjects for each variable; source data are provided as a Source Data file

**Supplementary Table 5. Characteristics of young healthy volunteers.**

| **Variables** | **ALL** | **MALES**  (n= 18) | **p** | **FEMALES (F)**  (n = 11) | **p** | **FEMALES (P)**  (n = 4) |
| --- | --- | --- | --- | --- | --- | --- |
| **Females** | **15 (45.5%)** | - |  | - |  | - |
| **Age** (years) | **25 ± 4** | 25 ± 3 | 0.769 | 25 ± 6 | 0.498 | 28 ± 5 |
| **Office SBP** (mmHg) | **115 ± 11** | 118 ± 12 | 0.096 | 111 ± 8 | 0.882 | 115 ± 8 |
| **Office DBP** (mmHg) | **65 ± 9** | 63 ± 10 | 0.501 | 66 ± 9 | 0.518 | 69 ± 8 |
| **Office HR** (beats per minute) | **67 ± 8** | 66 ± 10 | 0.318 | 69 ± 8 | 0.918 | 69 ± 4 |
| **BMI** (kg/m^2^) | **22.7 ± 2.8** | 23.0 ± 2.7 | 0.945 | 23.1 ± 3.0 | 0.109 | 20.4 ± 1.0 |
| **Body Weight** (Kg) | **68.3 ± 13.3** | 74.5 ± 13.0 | **0.012** | 62.1 ± 10.2 | 0.352 | 57.0 ± 3.9 |
| **Impedance** (whole body, Ω) | **630 ± 77** | 590 ± 68 | **0.0001** | 676 ± 33 | 0.891 | 682 ± 133 |
| **Total body water** (estimated; %) | **58.7 ± 6.0** | 62.4 ± 3.3 | **<0.0001** | 53.0 ± 5.2 | 0.131 | 58.0 ± 5.9 |
| **Total body fat** (estimated; %) | **19.7 ± 8.3** | 14.7 ± 4.5 | **<0.0001** | 27.7 ± 7.1 | 0.13 | 20.7 ± 8.1 |
| **Leg fat** (estimated; %) | **20.8 ± 9.9** | 13.2 ± 4.4 | **<0.0001** | 32.0 ± 3.9 | 0.098 | 27.2 ± 5.9 |
| **Trunk fat** (estimated; %) | **17.7 ± 7.1** | 15.3 ± 5.5 | **0.008** | 22.2 ± 6.8 | 0.255 | 16.7 ± 10.3 |
| **p-Na^+^** (mmol/l) | **141 (140-141)** | 141 (140-142) | 0.218 | 140 (140-141) | 0.376 | 140 (139-142) |
| **p-Urea** (mmol/l) | **4.7 ± 1.4** | 4.9 ± 1.7 | 0.336 | 4.5 ± 0.8 | 0.555 | 4.2 ± 1.0 |
| **p-Creatinine** (umol/l) | **76 ± 12** | 80 ± 11 | 0.111 | 72 ± 13 | 0.348 | 72 ± 13 |
| **24h-urinary volume** (ml) | **1875 ± 900** | 1800 ± 775 | 0.7 | 1950 ± 1150 | 0.879 | 2050 ± 775 |
| **24h-u Na excretion** (g/d) | **2.23 (1.77-3.27)** | 2.09 (1.47-3.52) | 0.555 | 2.37 (1.07-3.27) | 0.373 | 2.12 (1.89-2.48) |

Qualitative data presented as n (%) and compared by χ2 test. Quantitative data presented as mean ± SD or median (interquartile range) and compared by Student t-test or Mann-Whitney test, respectively, as appropriate. All tests are two-tailed. (F) = females in follicular phase; (P) = females in progestinic phase/state. BMI = Body Mass Index. SBP = systolic blood pressure. DBP = diastolic blood pressure. HR = heart rate. HTN = hypertension. Source data are provided as a Source Data file.

**Supplementary Figure 1. Blood pressure in the experimental groups.**

*Rats:* n=8 males/strain/intervention and n=10 females/strain/intervention, except n=9 female WKY on normal salt diet. *Panel a*: baseline systolic blood pressure (SBP) values before the experimental interventions, by sex and strain (n=16-20/group); data are presented as box-and-whisker plot (Tukey: center line, median; box limits, upper and lower quartiles; whiskers, 1.5x interquartile range; points, outliers). *Panel b*: SBP response to experimental intervention (change at 3 weeks vs baseline) by sex and strain. Data are presented as mean ± 95%CI and individual dots; X = automatically excluded outlier (ROUT, Q = 1%). For all panels, 2-way ANOVA results at bottom and post-hoc tests on top; # p < 0.05 for two-tailed one-sample t-test vs 0 mmHg (no change vs baseline; Female *WKY*_HS: p<0.001, Female *SHRSP*_HS: p=0.0147; Male *SHRSP*_HS: p<0.001). Source data are provided as a Source Data file.

**Supplementary Figure 2. Predicted and experimental histochemical results.**

**a**

**b**

*Panel a*: **X** = experimental results from control *WKY* rats in different tissues (means from Supplementary Data 1), superimposed on the general prediction model based on extracellular volume percentage (ECV%)^1^; red circles = predicted tissue [Na^+^], yellow squares = predicted tissue [K^+^]. As per Figure 1, the lower is relative tissue cellularity, the higher [Na^+^]. [Na^+^] and [K^+^] results effectively differentiate organs physiologically sitting at different points of the ECV/ICV scale. *Panel b, left:* percentage change of Na^+^ content (red bars) and water content (white bars) in tissues showing oedema accumulation upon HS in SHRSP rats (from Supplementary Data 1); *right*: same percentage change, normalised for water change, showing a proportionally larger increase in Na^+^ compared to water content when tissue cellularity is increasingly high, as predicted.^11^ Source data are provided as a Source Data file.

**Supplementary Figure 3. Fat content in skin and liver.**

*Panel a*: skin fat content, by strain and experimental treatment; 2-way ANOVA results at bottom and post-hoc inter-strain tests on top. Significant strain differences in skin fat content (approximately 3-5% of WW), regardless of treatment, are well in keeping with the difference in water (and Na^+^ content) observed between NS WKY and SHRSP (Figure 2a). *Panel b*: trends are similar for liver, although not significant; due to smaller content and relative difference (as for water content, Figure 2a) in liver compared to skin, this likely suggests a type II statistical error in rejecting a null hypothesis, rather than a lack of strain difference. Bars represent medians ± 95%CI, with individual dots (overlay; n=15-18/bar). Source data are provided as a Source Data file.

**Supplementary Figure 4. Water parallels Na^+^ excess.**

Regression lines for tissue Na^+^ and water content in different tissues. No difference in slope or intercept was detected across experimental groups in either organ, so that one curve adequately fitted all points. For skeletal muscle, no regression line was drawn, as no significant correlation was found within either group beforehand: the dissociation between Na excess and water in skeletal muscle likely reflects an oedema-independent shift in ECV/ICV ratio due to salt-induced catabolic muscle (cells) loss.^2^ For each tissue, n = 16-18 animals/group, as per figure 2 and Supplementary data 1; source data are provided as a Source Data file.

**Supplementary Figure 5. Myocardial Na^+^ excess, glycosaminoglycans content and TonEBP expression in hypertensive ageing.**

*Panel a*: bars show mean ± 95%CI for all rats (*WKY* 20w n=10; *WKY* 52w = 9; *SHRSP* 20w = 6; *SHRSP* 52w = 9). Myocardial Na^+^ content (and concentration) increased in 52 weeks vs 20 weeks old SHRSP, paralleled by water and mirrored by K^+^, so that tissue [Na^+^ + K^+^] was constant across groups (not shown); this is suggestive of myocardial oedema. *Panel b*: sulphated glycosaminoglycans (sGAG) content in mid-myocardium and sub-epicardium, estimated by Alcian Blue staining and normalised by WKY young rats (*WKY* 20w, *WKY* 52w = 4, *SHRSP* 20w = 5, *SHRSP* 52w = 4; ≥ 25 pictures of mid-myocardium and ≥ 12 pictures of sub-epicardium per animal); box-and-whisker plots (center line, median; box limits, upper and lower quartiles; whiskers, min-max); right inserts: representative Alcian Blue^+^ areas in mid-myocardium and sub-epicardium corresponding to dotted lines (i.e. 1x, 8x, 64x and 256x young WKY rats, as reference; black arrows: Alcian Blue staining; dotted lines inside inserts: epicardial border. Left ventricular sGAGs increased with ageing but was higher in SHRSP compared to WKY; whether this extracellular matrix remodelling facilitates myocardial oedema, is a consequence of it or both, in a sort of vicious circle, remains unknown. *Panel c*: data presented as Delta CT, median ± 95%CI and individual dots (overlay). Increased TonEBP gene expression followed oedema accumulation despite no hypertonicity. All tests are two-sided; panel a: two-sided Fisher LSD test, panel b-c: Mann-Whitney test. Source data are provided as a Source Data file.

**Supplementary Figure 6. Chemical analysis of aorta.**

Water, Na^+^ and K^+^ aortic content by sex, strain and experimental treatment; bars show median ± 95%CI; * p<0.05 for predefined two-tailed Mann-Whitney test, on top; X = automatically identified outliers. Although in line with previous reports,^3-4^ we acknowledge organ-specific technical limitations that should prompt cautious interpretations of these data: 1) chemical analysis of aortas was not performed in all animals and is therefore limited in power; 2) the large surface/size ratio of the tissue makes water evaporation extremely fast; the need to thaw the tissue at room temperature to strip out the periaortic fat layer at the time of sample cutting could impact on water (and overall tonicity, not shown) estimates; 3) intra-aortic blood was considered an unacceptable confounder and gently washed away with phosphate-buffered saline (PBS) at the time of organs harvesting; although devoid of K^+^ rich circulating blood cells, and despite the accurate blotting on paper after cutting the aortic rings transversally open, it may have impacted on the total Na^+^ content of the samples.

With the above limitations, data suggest an increase of water content in the aorta of SHRSP rats upon HS (with potential implications on stiffness), a higher K^+^ content in SHRSP rats (hypertensive smooth muscle cells hypertrophy?) and a trend for an increase in Na^+^ content in SHRSP-HS (possibly female-specific and suggestive of fibrosis and/or oedema). Source data are provided as a Source Data file.

**Supplementary Figure 7. Sex-differences in chemical composition of skin from young healthy volunteers and impact of subcutaneous fat.**

**
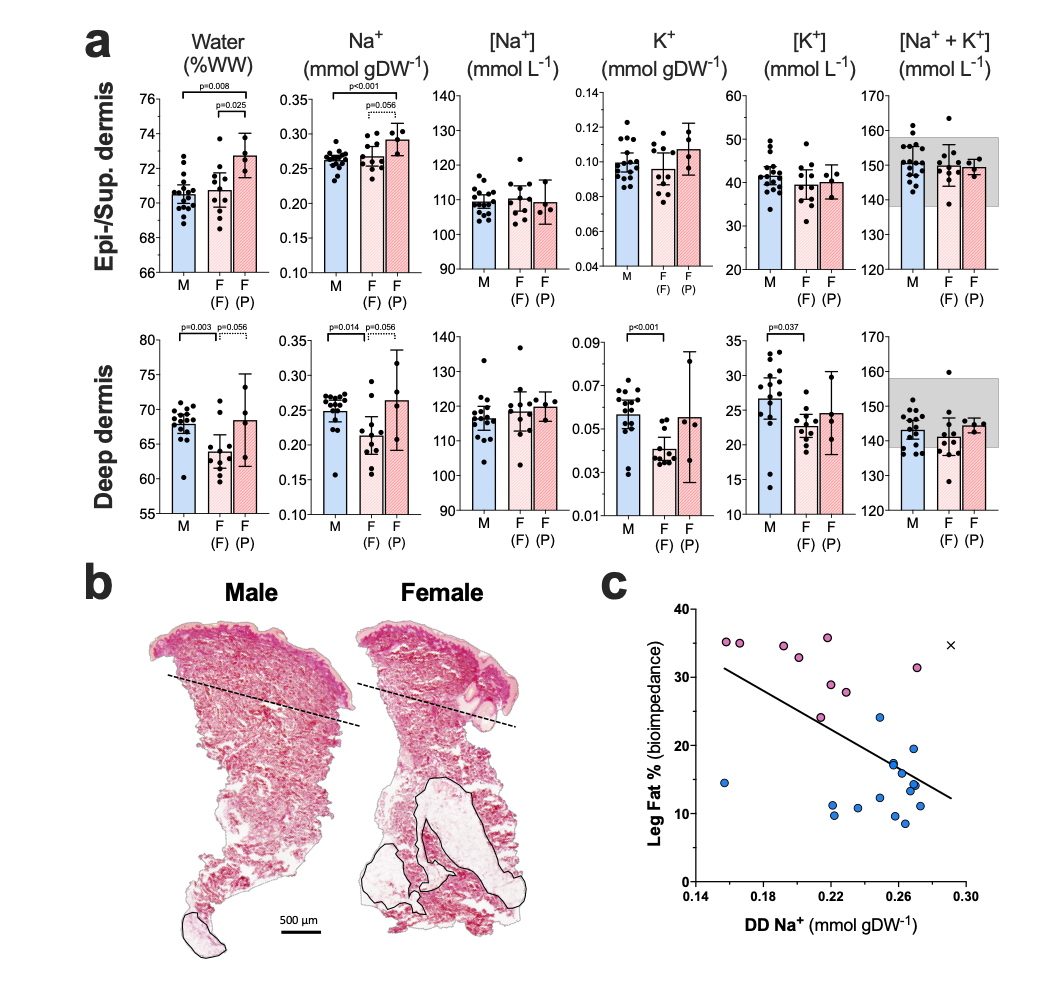
**

*Panel a*: mean ± 95%CI; blue (M) = males (n=18), light pink (F(F)) = females in follicular phase (n=11), dark pink (F(P)) = females in progestinic phase/state (n=4); group differences shown by brackets, at two-tailed Student t test. No differences were observed between M and F(F) in ESD. In DD, water, Na^+^ and K^+^ content, but not concentration, were lower in F(F) (p=0.003, p=0.014 and p=0.001, respectively). F(P) overall had higher skin water and Na^+^ content than F(F) (ESD: p=0.025 and p=0.056, respectively; DD: p= 0.056 for both). *Panel b*: representative skin sections from a male and a female subject (picrosirius-red staining; scale bar = 500 μm); dashed line = representative of the cutting plane for separation of ESD and DD in all study samples; black-circled areas in DD = subcutaneous fat, which is known to be more abundant in female subjects. *Panel c*: blue = M, pink = F(F), X = outlier (ROUT, Q = 1%). Inverse correlation (ρ = -0.54, p = 0.005) between gluteal dermal Na^+^ content and percentage fat mass in the leg, calculated by bioimpedance analysis; similar correlations were observed with directly-measured bioimpedance values (Ω; leg: ρ = -0.40, p = 0.047; whole-body: ρ = -0.62, p < 0.001) and for dermal water content (with leg fat %: ρ = -0.62, p = 0.001; with leg Ω: ρ = -0.42, p = 0.04; with whole-body Ω: ρ = -0.61, p < 0.001). These results suggest that subcutaneous fat limit the volume of distribution for both water and Na^+^ in the deep dermis, thus explaining sex differences in their DD content. Pearson ρ between Na^+^ and water content was > 0.9 (p < 0.0001), with no difference in the slope of the regression line between layers or sexes (p = 0.47 and 0.68, respectively). Source data are provided as a Source Data file.

**Supplementary Figure 8. Relationships between skin histochemical parameters and age, BMI and estimated Na^+^ intake.**

Univariate and multivariable-adjusted relationships, by skin layer; the multivariable model included age, sex, BMI and Na^+^ intake. Data presented as standardised B regression coefficients (95%CI); significant associations highlighted in red. All panels refer to a total of n=76 samples, with a maximum of n=8 pairwise-excluded missing values for each regression. Source data are provided as a Source Data file.

**Supplementary Figure 9. Reproducibility of tissue chemical analysis.**

Panel a: replication of the flame photometer chemical analysis on the same HNO_3_-digested samples (technical replicates; batch of female rat skin samples reanalyzed at time 2, months after time 1) shows excellent reproducibility (ρ = 0.98 [95%CI: 0.96 to 0.99]; p<0.0001). The shift from the identity line (in red; slope of the regression line = 0.91 [0.87-0.95]; p < 0.001 vs 1.0) is likely due to unavoidable minimal differences in the progressive dilutions of calibration standards at the two different times. To avoid any such systematic error, all samples from the same type of tissue were always analysed as a single batch on the same day; the few samples affected by experimental issues on that day could not be replicated and had to be excluded. Panels b-c: reproducibility of results from the same tissue of the same animal; random duplicate samples from different tissues are shown for water content (gravimetric approach; panel b) and Na content (flame photometry; panel c); red= identity line. Source data are provided as a Source Data file.

**SUPPLEMENTARY NOTE I - ImageJ MACRO for histological Alcian Blue^+^ area quantification**

run("Image Sequence...", "open=E:\\mic\\XXX\\untitled000.tif sort");

run("Invert", "stack");

run("Color Threshold...");

// Color Thresholder 1.50i

// Autogenerated macro, single images only!

min=newArray(3);

max=newArray(3);

filter=newArray(3);

a=getTitle();

run("HSB Stack");

run("Convert Stack to Images");

selectWindow("Hue");

rename("0");

selectWindow("Saturation");

rename("1");

selectWindow("Brightness");

rename("2");

min[0]=70;

max[0]=255;

filter[0]="stop";

min[1]=0;

max[1]=255;

filter[1]="pass";

min[2]=30;

max[2]=255;

filter[2]="pass";

for (i=0;i<3;i++){

selectWindow(""+i);

setThreshold(min[i], max[i]);

run("Convert to Mask");

if (filter[i]=="stop") run("Invert");

}

imageCalculator("AND create", "0","1");

imageCalculator("AND create", "Result of 0","2");

for (i=0;i<3;i++){

selectWindow(""+i);

close();

}

selectWindow("Result of 0");

close();

selectWindow("Result of Result of 0");

rename(a);

// Colour Thresholding-------------

run("Close");

run("Make Binary", "method=Default background=Light calculate list");

run("Image Sequence... ", "format=TIFF name=ANALISI save=E:\\mic\\analisi\\ANALISI0000.tif");

run("Measure...", "choose=E:\\mic\\analisi\\");

saveAs("Results", "E:\\mic\\analisi\\Results.xls");

close();

**SUPPLEMENTARY NOTE II – Food-frequency questionnaire**

**
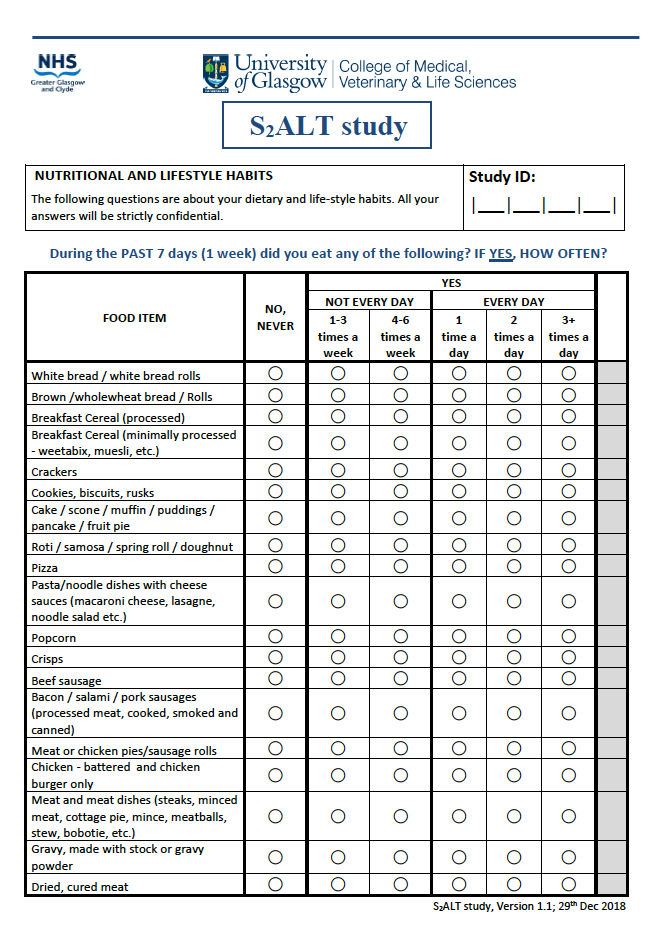
**

**
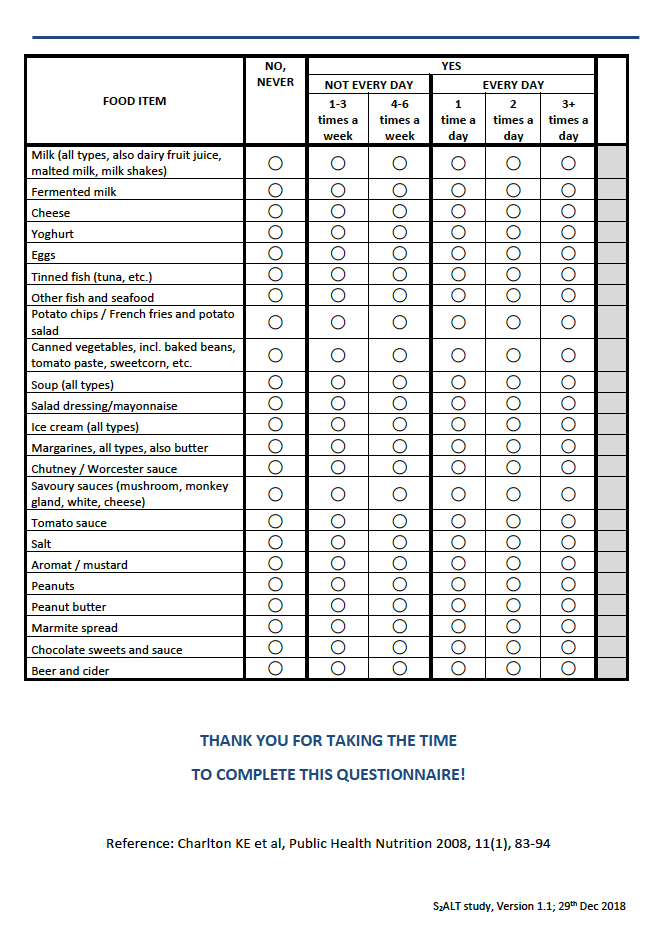
**

**SUPPLEMENTARY NOTE III - Figure 1 Credits**

The cell image used in Figure 1 was modified from the file ‘normal-cell-cancer-3’ from Servier Medical Art (<https://smart.servier.com/smart_image/normal-cell-cancer-3/>), licensed under the Creative Commons Attribution 3.0 Unported license.

**Supplementary references**

1. Rossitto G, Touyz RM, Petrie MC, Delles C. Much Ado about N...atrium: modelling tissue sodium as a highly sensitive marker of subclinical and localized oedema. *Clinical science (London, England : 1979)* **132**, 2609-2613 (2018).

2. Kitada K*, et al.* High salt intake reprioritizes osmolyte and energy metabolism for body fluid conservation. *The Journal of clinical investigation* **127**, 1944-1959 (2017).

3. Tobian L, Jr., Binion JT. Tissue cations and water in arterial hypertension. *Circulation* **5**, 754-758 (1952).

4. Tobian L, Janecek J, Tomboulian A, Ferreira D. Sodium and potassium in the walls of arterioles in experimental renal hypertension. *The Journal of clinical investigation* **40**, 1922-1925 (1961).
