# Supplementary material for: Tissue sodium excess is not hypertonic and reflects extracellular volume expansion
Source: Nat Commun. 2020 Aug 24;11:4222. doi: 10.1038/s41467-020-17820-2 (PMC7445299; doi:10.1038/s41467-020-17820-2)
Supplement: Supplementary file 3 — Description of Additional Supplementary Files [file 41467_2020_17820_MOESM3_ESM.docx]

**DESCRIPTION OF ADDITIONAL SUPPLEMENTARY FILES**

*File Name*: Supplementary Data 1
*Description*: Histochemical analysis of rat tissues. Data presented as mean ± SD. NS = normal salt, tap water; HS = high salt, 1% NaCl. Statistical tests noted on top, with all individual p values and summary symbols (*p<0.05, **p<0.01, ***p<0.001, ****p<0.0001). Source data are provided as a Source Data file.
