# Supplementary material for: Tissue sodium excess is not hypertonic and reflects extracellular volume expansion
Source: Nat Commun. 2020 Aug 24;11:4222. doi: 10.1038/s41467-020-17820-2 (PMC7445299; doi:10.1038/s41467-020-17820-2)
Supplement: Supplementary file 5 — Reporting Summary [file 41467_2020_17820_MOESM5_ESM.pdf]

nature research

Corresponding author(s): Giacomo Rossitto

Last updated by author(s): Jul 16, 2020

# Reporting Summary

Nature Research wishes to improve the reproducibility of the work that we publish. This form provides structure for consistency and transparency in reporting. For further information on Nature Research policies, see Authors & Referees and the Editorial Policy Checklist .

## Statistics

For all statistical analyses, confirm that the following items are present in the figure legend, table legend, main text, or Methods section.

| n/a | Confirmed | |
|---|---|---|
| ☐ | ☒ | The exact sample size (*n*) for each experimental group/condition, given as a discrete number and unit of measurement |
| ☐ | ☒ | A statement on whether measurements were taken from distinct samples or whether the same sample was measured repeatedly |
| ☐ | ☒ | The statistical test(s) used AND whether they are one- or two-sided<br>*Only common tests should be described solely by name; describe more complex techniques in the Methods section.* |
| ☐ | ☒ | A description of all covariates tested |
| ☐ | ☒ | A description of any assumptions or corrections, such as tests of normality and adjustment for multiple comparisons |
| ☐ | ☒ | A full description of the statistical parameters including central tendency (e.g. means) or other basic estimates (e.g. regression coefficient) AND variation (e.g. standard deviation) or associated estimates of uncertainty (e.g. confidence intervals) |
| ☐ | ☒ | For null hypothesis testing, the test statistic (e.g. *F*, *t*, *r*) with confidence intervals, effect sizes, degrees of freedom and *P* value noted<br>*Give P values as exact values whenever suitable.* |
| ☒ | ☐ | For Bayesian analysis, information on the choice of priors and Markov chain Monte Carlo settings |
| ☒ | ☐ | For hierarchical and complex designs, identification of the appropriate level for tests and full reporting of outcomes |
| ☐ | ☒ | Estimates of effect sizes (e.g. Cohen's *d*, Pearson's *r*), indicating how they were calculated |

*Our web collection on statistics for biologists contains articles on many of the points above.*

## Software and code

Policy information about availability of computer code

| | |
|---|---|
| Data collection | Dedicated tail-cuff SBP measurement software (rat blood pressure); Olympus BX41 microscope image capture software and ImageJ (v. 1.52) for histology analysis; Labchart (ADinstruments, v. 7; for vascular function studies on wire myograph); Courage & Khazaka proprietary software provided with Tewameter TM300 (transepidermal water loss; https://www.enviroderm.co.uk/products/tewameter-tm-300); Microsoft Excel (v. 16) for all data collection. |
| Data analysis | Prism (GraphPad Software, version 8) and SPSS (IBM, version 25) |

For manuscripts utilizing custom algorithms or software that are central to the research but not yet described in published literature, software must be made available to editors/reviewers. We strongly encourage code deposition in a community repository (e.g. GitHub). See the Nature Research guidelines for submitting code & software for further information.

## Data

Policy information about availability of data

All manuscripts must include a data availability statement. This statement should provide the following information, where applicable:

- Accession codes, unique identifiers, or web links for publicly available datasets
- A list of figures that have associated raw data
- A description of any restrictions on data availability

all data supporting the findings of this study are available within the paper and its supplementary information files (source data file)

# Field-specific reporting

Please select the one below that is the best fit for your research. If you are not sure, read the appropriate sections before making your selection.

# Life sciences study design

All studies must disclose on these points even when the disclosure is negative.

| | |
|---|---|
| Sample size | For animal studies, the sample size and n/group was based on similar experiments conducted by us or others (refs 16-18) in the same or similar rat strains.<br>For the human study, as per REC approval, no formal sample size calculation was performed because of the cross-sectional nature of the design and the descriptive/correlation statistics planned. Based on the number of patients attending the High Blood Pressure Clinic and the estimated rate of consent to participation, we planned to include approximately 150 participants. At the end of the timeframe available (Carnegie Trust Scholarship to JYC) we recruited 90 subjects, of whom 76 (84%) agreed to undergo also the skin biopsy: the lower overall recruitment was balanced by a much higher rate of consent to the invasive optional part of the study than initially anticipated. The final sample size gave us sufficient power to address the primary and most of the secondary study questions and to include a statistically legitimate number of relevant covariates in the regression models to explore independent associations. |
| Data exclusions | As per methods section:<br>- for tissue chemical nalysis, all samples from the same type of tissue were analysed in a batch on the same day to minimise technical variability; therefore, few samples irreversibly affected by experimental issues (e.g. sample fall, contamination, volume shortage) had to be excluded and are reported as blank cells in the Source Data File (pre-stablished criterion)<br>- vessels with an internal diameter larger than what traditionally considered typical for resistance arteries (AM Heagerty, Hypertension 1993) or lacking viable responses were excluded (pre-stablished criterion)<br>- handling of missing data for the salt intake questionnaire was not pre-established. However, we post-hoc verified that data were missing at random (Little's MCAR test, $p = 0.593$); 5 subjects with more than half of the responses missing were excluded from calculation of weekly scores; 22 additional subjects had ≤10% missing responses (10/22 had only 1 missing item): median substitution was used for imputation and calculation of weekly scores; results without imputation were overall identical.<br>- pre-defined automatic outliers detection was conducted by ROUT method (Q = 1%; Prism, GraphPad Software, version 8). |
| Replication | For the tissue chemical analysis, i.e. water and Na+/K+ measurements, we optimised the protocol and verified the reproducibility of our method prior to all experiments presented in this manuscript, by using tissues from other rats (both WKY and SHRSP). At the calibration used, reported CV% for reproducibility is < 2%; in our hands it was 1.5% for repeated measures on the same sample (for Na+/K+) and 3% for measures on independent samples from the same tissue of the same animal.<br>The surgical time for dissection of tissues at the time of culling was deliberately minimised to prevent evaporation of moisture, seen as a possible relevant source of bias in previous independent investigations by others (e.g. whole body de-skinning). Since additional pieces of the same tissues were also stored for molecular biology studies, this left us with limited amount of tissue for replication of chemical analysis. This is particularly true also for human skin samples from punch biopsies, deliberately kept to a size which was suitable for a single tissue drying, digestion and photometer analysis process, but also minimally invasive, without the need for stitches.<br>Nevertheless, we further verified reproducibility of our analysis during the study experiments by:<br>- Replicating the measurements on stored left-over material from previously analysed batches of digested samples. For technical reasons (complete sample use) this was not feasible for tissue water and fat content analysis.<br>- Processing, as part of an animal tissue batch, some duplicate samples from the same animal, with the only criterion of adequate tissue availability from that specific animal in the random sample selection.<br>Results of these checks are presented in Suppl Fig 9. All samples from the same type of tissue were analysed in a batch on the same day to minimise any systematic error due to unavoidable minimal differences in the progressive dilutions of calibration standards.<br>TonEBP gene expression analysis was not replicated but conducted in duplicate.<br>Due to design complexity, number of animals and availability of myograph baths, vascular function analyses under different experimental conditions were performed on a single vessel/experimental condition per animal.<br>Transepidermal Water Loss was measured only once per patient, in the interest of study visit duration. |
| Randomization | Rat littermates were randomly allocated to in-vivo experimental conditions (methods, first section). Similarly, 2 mm arterial segments from third order mesenteric branches were randomly allocated to experimental ex-vivo conditions in the myograph baths. Randomisation does not apply to the human study because of its non-interventional nature. |
| Blinding | Rat systolic blood pressure was measured by tail-cuff plethysmography, in an operator-blind fashion whenever possible (e.g. when there were no obvious differences in body weight and/or behaviour and or hair between single rats that made it obvious to the person taking the measurement). During all tissue processing, flame photometry, vascular function studies and generation of all final datasets, operators/ researchers were blind to group allocation of samples. |

# Reporting for specific materials, systems and methods

We require information from authors about some types of materials, experimental systems and methods used in many studies. Here, indicate whether each material, system or method listed is relevant to your study. If you are not sure if a list item applies to your research, read the appropriate section before selecting a response.

## Materials & experimental systems

| n/a | Involved in the study | |
|---|---|---|
| x | | Antibodies |
| x | | Eukaryotic cell lines |
| x | | Palaeontology |
| | x | Animals and other organisms |
| | x | Human research participants |
| | x | Clinical data |

## Methods

| n/a | Involved in the study | |
|---|---|---|
| x | | ChIP-seq |
| x | | Flow cytometry |
| x | | MRI-based neuroimaging |

# Animals and other organisms

Policy information about studies involving animals; ARRIVE guidelines recommended for reporting animal research

**Laboratory animals**

We used stroke-prone spontaneously hypertensive (SHRSP) and Wistar-Kyoto (WKY) rats from colonies inbred at the University of Glasgow since 1991. Main data (salt loading): male and female rats (n=8-10/sex/experimental group) treated from 12th to end of 14th week of age; Supplementary data (ageing): male rats maintained on standard diet and normal tap water ad libitum until 20 and 52 weeks of age (n = 6-10/age/group)

**Wild animals**

The study did not involve wild animals

**Field-collected samples**

the study did not involve samples collected from the field.

**Ethics oversight**

All animal protocols were approved by the institutional ethics review committee (University Animal Welfare and Ethics Review Board).

Note that full information on the approval of the study protocol must also be provided in the manuscript.

# Human research participants

Policy information about studies involving human research participants

**Population characteristics**

The hypertensive subjects participating in the S2ALT study were representative of both sexes (47.4% females) and of a broad age range (22-86; mean ± SD = 58 ± 15 years old). Blood pressure was overall uncontrolled (148 ± 21/89 ± 12 mmHg), despite a median [interquartile range] of 2 [0-4] antihypertensive medications, including an ACE inhibitor or an ARB in almost 8-% of the cases. There was a high prevalence of obesity and dyslipidaemia (>50% for both) and a relatively low prevalence of diabetes or chronic kidney disease (<15% for both).

The young (25 ± 4 years old) healthy subjects participating in the SOWAS study were predominantly males (18/33); out of 15 females, 11 were on a follicular phase of their menstrual cycle. They were all normotensive (115 ± 11/65 ± 9 mmHg), had an average-low Na+ consumption, as estimated by 24h-u Na excretion (2.23 [1.77-3.27] g/d) and had no history of cardiovascular, metabolic or renal disease.

**Recruitment**

For S2ALT study, all adult patients scheduled for an outpatient appointment at Glasgow HBP Clinic received a Patient Information Leaflet (PIL) by post to 7-10 days before they attended, along with the usual appointment confirmation letter, for their consideration. Consecutive, non-pregnant, unselected patients providing valid written informed consent on occasion of the Clinic appointment underwent the study exams/procedures on the same day ,with no incentive apart from their generous and altruistic participation to research. In light of the general profile of the study population (please see above), highly representative of the typical hypertensive patients attending a Hypertension Clinic, we do not foresee any obvious source of bias with biological impact.
The small young healthy volunteers cohort was recruited among University students by advertisement, with a 30£ voucher as a compensation for time and potential travel expenses. This may have generated a selection bias, but overall does not appear to have a biological impact on the skin biological characteristics of our study population.

**Ethics oversight**

The protocol for the S2ALT (Skin Sodium Accumulation and water baLance in hypertension) study was approved by the West of Scotland Research Ethics Committee 3 (ref. 18/WS/0238) and Greater Glasgow and Clyde NHS Research and Development (ref. GN18CA634). The recruitment of the small young healthy volunteers cohort (supplementary data) was approved by the University of Glasgow, MVLS College Ethics Committee (ref. 8200170153).

Note that full information on the approval of the study protocol must also be provided in the manuscript.

# Clinical data

Policy information about clinical studies

All manuscripts should comply with the ICMJEguidelines for publication of clinical research and a completedCONSORT checklist must be included with all submissions.

**Clinical trial registration**

not applicable

| | |
|---|---|
| Study protocol | not applicable. All the protocol relevant details are reported in the Methods section and/or this summary. |
| Data collection | Adult, non-pregnant patients were recruited from the High Blood Pressure clinic, Queen Elizabeth University Hospital, Glasgow between March and July 2019. On the occasion of their scheduled clinic appointment (9:00 AM to 4:30 PM), they underwent the study exams/procedures in the same site. |
| Outcomes | Primary: histochemical characterization of skin biopsies (water, Na+ and K+ content/concentration) - also in relation to local mechanisms of Na/water homeostasis if Na were shown as hypertonic.<br>Assessment: tissue chemical analysis; transepidemal water loss; sweat chemical analysis (not reported here)<br><br>Secondary: association between histochemical measures and clinical characteristics of patients<br>Assessment: collection of routine clinical parameters; salt-intake validated questionnaire. |
